# Protective Effect of Quercetin and Ginger (*Zingiber officinale*) Extract against Dimethoate Potentiated Fluoride-Induced Nephrotoxicity in Rats

**DOI:** 10.3390/foods12091899

**Published:** 2023-05-05

**Authors:** Priyanka Sharma, Pawan Kumar Verma, Shilpa Sood, Rasia Yousuf, Amit Kumar, Rajinder Raina, Muhammad Asim Shabbir, Zuhaib F. Bhat

**Affiliations:** 1 Division of Veterinary Pharmacology and Toxicology, Faculty of Veterinary Science and Animal Husbandry, SKUAST-Jammu, Jammu 181102, India; 2 Division of Veterinary Pathology, Faculty of Veterinary Science and Animal Husbandry, SKUAST-Jammu, Jammu 181102, India; 3 Quality Management and Instrumentation Division, Indian Institute of Integrative Medicine (CSIR-Lab), Jammu 180016, India; 4 National Institute of Food Science and Technology, University of Agriculture, Faisalabad 38000, Pakistan; 5 Division of Livestock Products Technology, SKUAST-Jammu, Jammu 181102, India

**Keywords:** fluoride, dimethoate, nephrotoxicity, quercetin, *Zingiber officinale*, Wistar rats

## Abstract

This study aimed to determine the potential of quercetin and *Zingiber officinale* (ZO) Roscoe extract to alleviate the renal damage induced by dimethoate (DM) and fluoride (F^-^) alone and by their combined exposure in rats. A total of 54 adult Wistar rats were randomly allocated to nine groups (*n* = 6). A sub-lethal dose of DM (1/10th of the median lethal dose) was administered by oral gavage alone and along with F^-^ (4.5 ppm, three-fold the permissible limit) in their drinking water continuously for 28 days. Chromatographical analysis revealed the presence of quercetin, curcumin, and other phytochemicals with strong antioxidant properties in ZO-rhizome extract. Severe changes were observed in the levels of the renal biomarkers and histoarchitecture after co-administration of the toxicants, indicating greater kidney damage. The administration of ZO extract (300 mg/kg) along with either or both toxicants led to a significant restoration of the biochemical markers and renal antioxidant profile and histology.

## 1. Introduction

Rapid industrialization has reduced the cultivable land area, which has led to an indiscriminate application of pesticides to increase food production for a rapidly growing population, especially in the developing world. This has introduced a significant volume of pollutants to the environment and aquatic ecosystems. Dimethoate (DM, *O*,*O*-dimethyl-S-methylcarbamoylmethyl phosphorothioate), an organophosphorus compound, is commonly used in agriculture as an insecticide [1,2] to control the arthropod infestation of agricultural products [3]. DM has been classified as a class II agent by WHO and is a well-known neurotoxin that inhibits acetylcholinesterase (AChE), interrupts cholinergic neurotransmission in the central and peripheral nervous systems, and alters metabolic enzymes in target and non-target species, including mammals [2]. Continuous exposure to low levels of DM induces oxidative stress and DNA damage and impairs the functioning of membrane-integrated ATPases, which can lead to the compromised functioning of visceral organs, especially the kidney [4].

Fluorine is another pollutant that is highly abundant in air, water, and soil due to anthropogenic and geogenic activities, and its consumption results in fluorosis, a crippling disease that affects inhabitants in most parts of the globe. After exposure, fluoride (F^-^) is readily absorbed in the body and reaches various organs such as the heart, kidney, muscle, liver, bone, and brain, and impairs tissue function by inducing oxidative damage [5,6]. The exact mechanism of F^-^ toxicosis is not known, but it has been suggested that oxidative stress plays an important role in F^-^-mediated damage to various tissues. It binds with calcium ions, induces hypocalcemia, and causes the disruption of various physiological processes, leading to cardiovascular impairment, developmental and neurobehavioral disorders, and chronic renal diseases [7,8]. It also affects pro-oxidative capabilities and inhibits antioxidant enzymes, which are at the forefront in fighting oxidative stress. Furthermore, F^-^ exposure impairs AChE activities, which may augment organophosphorus-induced multi-systemic toxicity [9,10]. Previous studies have established an association between low levels of F^-^ exposure and significant alterations in hepatorenal health indices of humans and animals [11,12,13].

In the contemporary scenario, animals and human populations are exposed to different toxicants simultaneously, and some of them may act together to cause nephrotoxicity. Several studies have revealed that co-exposure to more than one environmental pollutant results in additive or synergistic toxicity [14,15,16]. Therefore, the high and ever-rising environmental contamination of DM along with naturally elevated F^-^ levels in the groundwater poses an increased risk of renal toxicity to exposed people or animals. The effect of combined toxicity can be exceptionally deleterious to people with pre-existing compromised renal functioning, such as those suffering from chronic renal diseases. It can also be a serious occupational hazard for people working in endemic fluorosis areas and employed in the pesticide application business [17].

New sources of dietary antioxidants are studied for their nephroprotection potential and are used clinically for relieving stress in patients with kidney impairment [17]. Studies have demonstrated that the intake of herbal extracts imparts protection against oxidative damage to kidneys by DM [18] and is beneficial in reducing fluoride toxicity [19]. However, the efficacy of ginger in ameliorating the combined toxicosis of F^-^ and DM has not been elucidated yet and needs scientific attention. The aim of this study was to evaluate the ameliorative antioxidant potential of ginger extract against nephrotoxicity induced by subacute exposure to F^-^ and DM alone and their concurrent exposure in Wistar rats.

## 2. Materials and Methods

### 2.1. Preparation of Zingiber officinale (ZO) Roscoe Extract

The rhizomes of ZO were purchased from the local market and identified by the taxonomists of the University of Kashmir (Voucher specimen No. 2921-(KASH) Herbarium, Centre for Biodiversity and Taxonomy, University of Kashmir, India). The rhizomes were cleaned, dried, and pulverized into a fine powder using an electric grinder (Usha MG 2053E Optima Mixer Grinder, Haryana, India). The grounded powder was sieved through muslin cloth which yielded a fine powder of particle size ranging between 2 and 14 µm. Powdered ZO was subjected to hydroalcoholic extraction (1:1 *v*/*v*, 4–5 h) in the Soxhlet apparatus, keeping the hot plate temperature between 65 and 70 °C. The extraction was repeated three times, and the extract was finally dried in a rotatory evaporator (55–60 °C, 15 rpm) and stored in a glass jar for further use [20].

### 2.2. Instrumentation and Chromatographical Analysis

The analysis was performed using Shimadzu HPLC consisting of a quaternary pump and photodiode array (PDA) detector and an autosampler. The analytical column used was the RP-18E column with the following dimensions: 5 μm, 250 mm × 4 mm. The mobile phase consisted of acetonitrile (Solvent A) and 0.1% acetic acid in water (Solvent B), and the separation was made using binary gradient elution. The column oven temperature used was 35 °C. The flow rate used was 0.8 mL/min, the injection volume was 10 µL, and the detection wavelength was 370 nm for the extract. Curcumin and quercetin were selected for the standardization of the extract. Standardization of the high-performance liquid chromatographical (HPLC) method (mobile phase, flow rate, and column temperature) including the ratio of acetonitrile and water containing acetic acid is presented in Table 1.

### 2.3. Experimental Design

The in vivo experimental trial was conducted on healthy Wistar rats for a period of 28 days. Animals of either sex weighing 180–190 g were purchased from the Indian Institute of Integrative Medicine (CSIR Lab), Jammu, India. The animals were raised under standard managemental conditions (25 ± 2 °C temp, 50 ± 15% relative humidity and normal photoperiod (12 h light–dark cycle)). All the animals were provided pelleted rations and clean drinking water ad libitum. The rats were acclimatized to the laboratory conditions for a period of three weeks prior to the start of experimentation and were under constant observation during the entire study period. The experimental protocols were duly approved by the Institutional Animal Ethics Committee (IAEC) vide proposal No. 3/IAEC/2020 Dated 22 October 2020 (Registration No. of IAEC–862/ac/04/CPCSEA). All experimental animals received humane care in accordance with the National Institute of Health Guide for the Care and Use of Laboratory Animals (NIH Publication No. 85-23, revised 1996). Dimethoate (DM) was administered by oral gavage at the dose rate of 31.0 mg/kg BW (body weight) (1/10th dose of median lethal dose (LD_50_)) [21]. Sodium fluoride was used as a source of fluoride at the concentration of 4.5 ppm (9.945 mg/L, three times the permissible limit set by WHO) in drinking water [6,7]. A total of fifty-four (54) adult Wistar rats were randomly allocated to nine groups (*n* = 6), and details of the treatment regimen followed for 28 days are presented in Table 2.

The levels of renal plasma biomarkers, viz., blood urea nitrogen (BUN), creatinine (CR), and uric acid (UA) were used to confirm the renal damage on exposure to toxicants. The results of the preliminary trials and the previous literature were used to determine the level of ZO (300 mg/kg BW).

### 2.4. Sample Collection and Analysis

At the end of the experimental trial, animals were sacrificed by cervical dislocation, 3–4 mL of heart blood was collected from each animal in heparinized tubes, and kidneys were collected in ice-cold 0.5 M phosphate buffer (pH 7.4) for antioxidant biomarker studies and in 10% formalin for histopathological examination. Tissue homogenate (10%) was prepared by homogenizing the tissue using a Teflon-coated homogenizer at 1000 rpm for 5–7 min at 4 °C. Whole blood was used for the estimation of hemoglobin and reduced glutathione (GSH) levels. Plasma for the biochemical analysis was obtained after the centrifugation of blood at 3000 rpm for 15 min and was kept at 4 °C for further analysis. Plasma renal biomarkers, viz., BUN, CR, UA, protein profile (total plasma proteins and albumin), and minerals (calcium and phosphorus) were determined using standard kits supplied by Transasia Bio-Medicals Ltd., India, using Chemistry Analyzer (CHEM-7, Mannheim, Germany). Levels of nitric oxide (NO) were measured in the plasma spectrophotometrically using a copper–cadmium alloy [22]. The principle of the assay is the reduction in nitrate to nitrite by the copper–cadmium alloy, followed by color development on the addition of a Griess reagent (sulfanilamide and N-naphthyl ethylenediamine) in an acidic medium, which was measured spectrophotometrically at 545 nm.

### 2.5. Fluoride Estimation

The fluoride level was estimated (*w*/*v* wet basis) in plasma and kidneys according to the standard extraction method [23]. In brief, 1.0 g of fresh tissue was homogenized in a solution containing 6.25 mL of 5.0% solution of sodium versanate and 6.25 mL of total ionic strength adjuster buffer III (TISAB III). Thereafter, the sample container was kept in a shaking water bath at 95 °C for 15 min. Cooled samples were filtered in a suction funnel, and filtrate was reconstituted to 25 mL with distilled water for estimation of fluoride using ion-selective electrochemistry (ISE) analyzer (Versa Star Pro Benchtop Electrochemistry meter, Thermo Scientific Orion, Waltham, MA, USA). The ISE was first calibrated with working standard fluoride ion concentrations in spiked plasma and tissue samples. The recovery of standard fluoride ranged between 89 and 92% during the standardization. The lower detection limit of equipment was 0.025 ppm, and a calibration curve was prepared using different concentrations (0.05, 0.50 and 5.0 ppm) for the determination of fluoride levels in the renal tissue [23]. The average slope of the standard curve used for the estimation was −58.3 mV/dec.

### 2.6. Determination of Antioxidant Biomarkers in Renal Tissue

The activity of arylesterase (AE) was measured using phenyl acetate as a substrate and was expressed in units per mL (U/mL), where one unit corresponds to μmol phenol formed per min [24]. Reduced glutathione (GSH), total antioxidant status (TAS), and total thiols (TTH) were estimated using standard methods [25,26,27]. The enzymatic component of antioxidant parameters, viz., catalase (CAT) and glutathione peroxidase (GPx) were determined using standard methods [28,29]. The activities of superoxide dismutase (SOD) and glutathione reductase (GR) were determined following the method described by Marklund and Marklund [30] and Carlberg and Mannervik [31], respectively. Similarly, malondialdehyde (MDA) and advance oxidation protein product (AOPP) levels in renal tissue were determined using standard methods [32,33], respectively.

### 2.7. Histopathology

Representative samples of kidneys from different groups were collected in formal saline and were washed, dehydrated, cleared, and embedded in paraffin, and then sectioned and stained with hematoxylin and eosin. The prepared sections were examined for the presence of histomorphological changes. The microscopic lesions were scored as (−), (+), (++), and (+++), for no, mild, moderate, and severe changes in the renal tissue, respectively.

### 2.8. Statistical Analysis

The data (*n* = 6) are presented as mean ± standard error and were analyzed by ANOVA using the Duncan Multiple Range tests and post hoc TUKEY LSD ALPHA at a 5% level of significance (0.05) using SPSS 21.0.

## 3. Results

### 3.1. Chromatographical Analysis of the ZO Rhizome Extract

The standardization of the high-performance liquid chromatographical (HPLC) method (mobile phase, flow rate, and column temperature) including the ratio of acetonitrile and water containing acetic acid was thoroughly studied (Table 2). The binary gradient method resulted in the appropriate separation of the extract and other active markers including curcumin and quercetin. Chromatograms of the extract and the standard compounds (curcumin and quercetin) are presented in the Supplementary file (Figure S1).

### 3.2. Fluoride Levels in the Plasma and Renal Tissues

**Figure 1:**
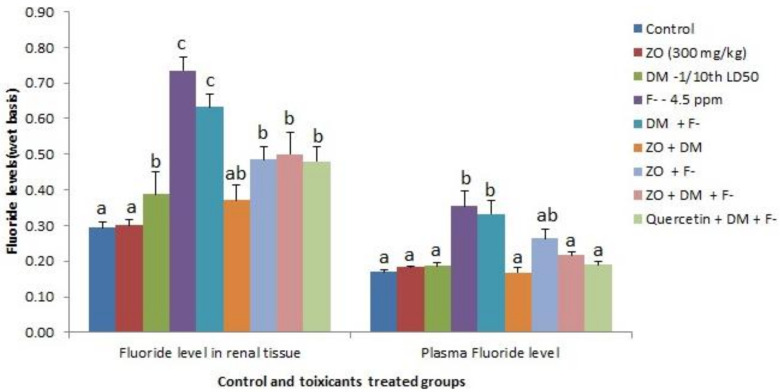
Alterations in the fluoride levels of plasma and renal tissues (% wet basis) of Wistar rats following subacute exposure to fluoride (F^-^) and dimethoate (DM) alone and in combination with hydroalcoholic extract of *Zingiber officinale* (ZO) (Values with different superscripts (a, b, c) on columns are statistically different from one an-other at 5% level of significance).

The feed and drinking water provided to the animals were subjected to fluoride estimation and contained 1.39 ± 0.21 ppm (% dry matter basis) and 1.34 ± 0.17 ppm, respectively. The fluoride levels in the plasma and renal tissues (% wet basis) of the Wistar rats in different groups are presented in Figure 1. The fluoride levels in the plasma and renal tissues of the control and different toxicant-exposed animals were determined, and the highest levels were recorded in group IV for both plasma and renal tissues. However, the supplementation with ZO or quercetin reduced the spike in fluoride levels observed in both plasma and kidneys in toxicant-only treatment groups receiving F^-^.

### 3.3. Plasma and Renal Biomarkers

The mean values of BUN, CR, and UA of the control and different treatment groups are presented in Table 3. A significant rise in BUN and CR was seen in the animals exposed to DM or F^-^ alone (groups III and IV, respectively) compared to the control. The rats administered with both the toxicants (group V), however, showed significantly higher BUN and CR values compared to the rats receiving only one toxicant. While the administration of ZO significantly decreased the BUN and CR in rats receiving F^-^ or DM alone, the co-administration of ZO completely reversed the CR values in the dual toxicant group; however, only partial amelioration occurred in the case of BUN. Both ZO and quercetin were equally effective in improving the combined effect of F^-^ and DM on the BUN and CR levels. The UA levels were found elevated in animals exposed to DM or F^-^ alone or when combined; however, the levels in the dual toxicant group were not significantly higher than those of the DM alone group. While the addition of ZO completely prevented any change in UA levels in single or dual toxicant groups, quercetin provided protection to dual toxicant-exposed rats.

Changes observed in plasma calcium (Ca) and phosphorus (P) levels in different experimental rats are presented in Table 3. The Ca and P levels showed a significant drop in all the groups exposed to toxicants alone compared to the control animals, and no further decrease was observed when both the toxicants were administered together. Both ZO and quercetin were effective in normalizing the plasma concentration of Ca and P in all the toxicant groups.

### 3.4. Effect on Plasma GSH and NO

**Figure 2:**
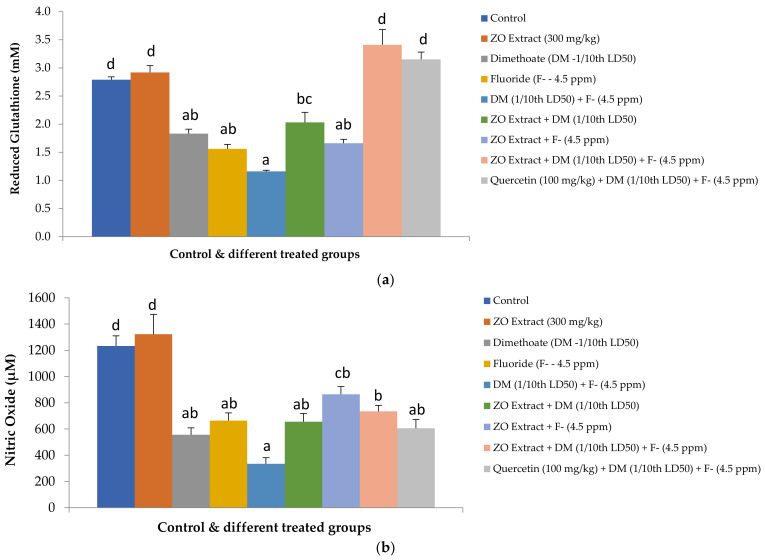
Effect of hydroalcoholic extract of *Zingiber officinale* (ZO) on levels of reduced glutathione (**a**) and nitric oxide (**b**) in plasma of rats following subacute exposure of fluoride (F^-^) and dimethoate (DM) alone and in combination (values with different superscripts (a, b, c, d) on columns are statistically different from one another at 5% level of significance).

Plasma GSH and NO levels were significantly reduced in all the groups administered with toxicants alone or in combination (groups III, IV, and V) compared to the control group (Figure 2). Both GSH and NO levels in group V were significantly lower compared to the control. The administration of ZO increased the levels of both NO and GSH in animals exposed to toxicants, whether alone or in combination, but the levels were significantly higher between groups V and VIII. Quercetin also improved GSH (*p* < 0.05) and NO (*p* > 0.05) levels in group IX compared to group V. However, ZO was more effective in increasing NO (group VIII, *p* < 0.05) compared to quercetin (group IX, *p* > 0.05), indicating the better protection of ZO in dual toxicant-exposed animals. Overall, the herbal agents could not restore the NO level completely, but the level of GSH became comparable to that of the control.

### 3.5. Antioxidant Biomarkers in Renal Tissue

Table 4 represents the data of various antioxidant enzymes in the renal tissue of control and treated groups. After exposure to toxicants either alone or in combination (groups III, IV, and V), the levels of TAS, TTH, AE, and AChE were significantly decreased compared to the control. A significantly greater decrease was observed in the levels of all these enzymes in rats after concurrent exposure to F and DM compared to animals given either F or DM alone. The administration of ZO to rats administered individual toxicants (groups IV and VII) significantly increased the levels of AChE and AE; however, the TAS and TTH levels in groups VI and VII did not improve compared to those of groups III and IV. The administration of ZO completely restored the levels of TAS, TTH, AE, and AChE in animals exposed to both toxicants simultaneously, whereas quercetin could not completely restore the AE and TTH levels. The CAT levels were significantly affected only in animals exposed to both the toxicants, and both ZO and quercetin supplementation significantly restored the levels. Significant reductions in SOD, GPx, and GR levels and a significant increase in AOPP and MDA levels were observed in all the groups administered with individual toxicants compared to control rats (Table 5).

The changes in SOD, AOPP, and MDA were more intense in the groups exposed to combined toxicants. The treatment with ZO reversed the changes in the levels of AOPP and MDA and GPx, SOD, and GR in all groups exposed to toxicants. Similar results were observed for quercetin co-treatment. While quercetin was more effective than ZO in ameliorating MDA levels, it was less so in the case of GR levels.

### 3.6. Histopathological Alterations in Renal Tissue

**Figure 3:**
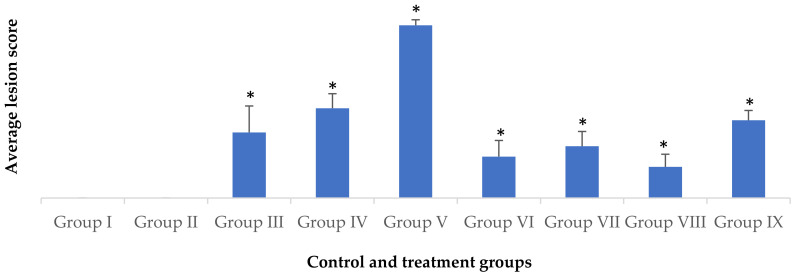
Average histopathological lesion score of renal tissues in control and treated groups (Columns with different * are statistically different from one another at 5% significance).

**Figure 4:**
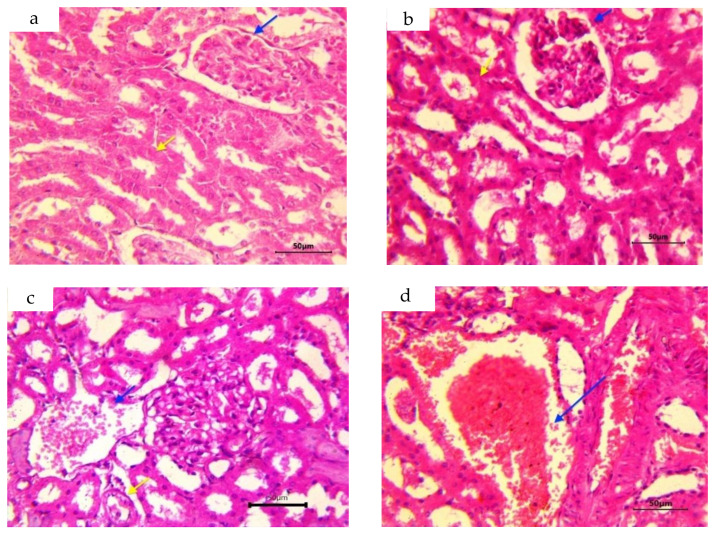

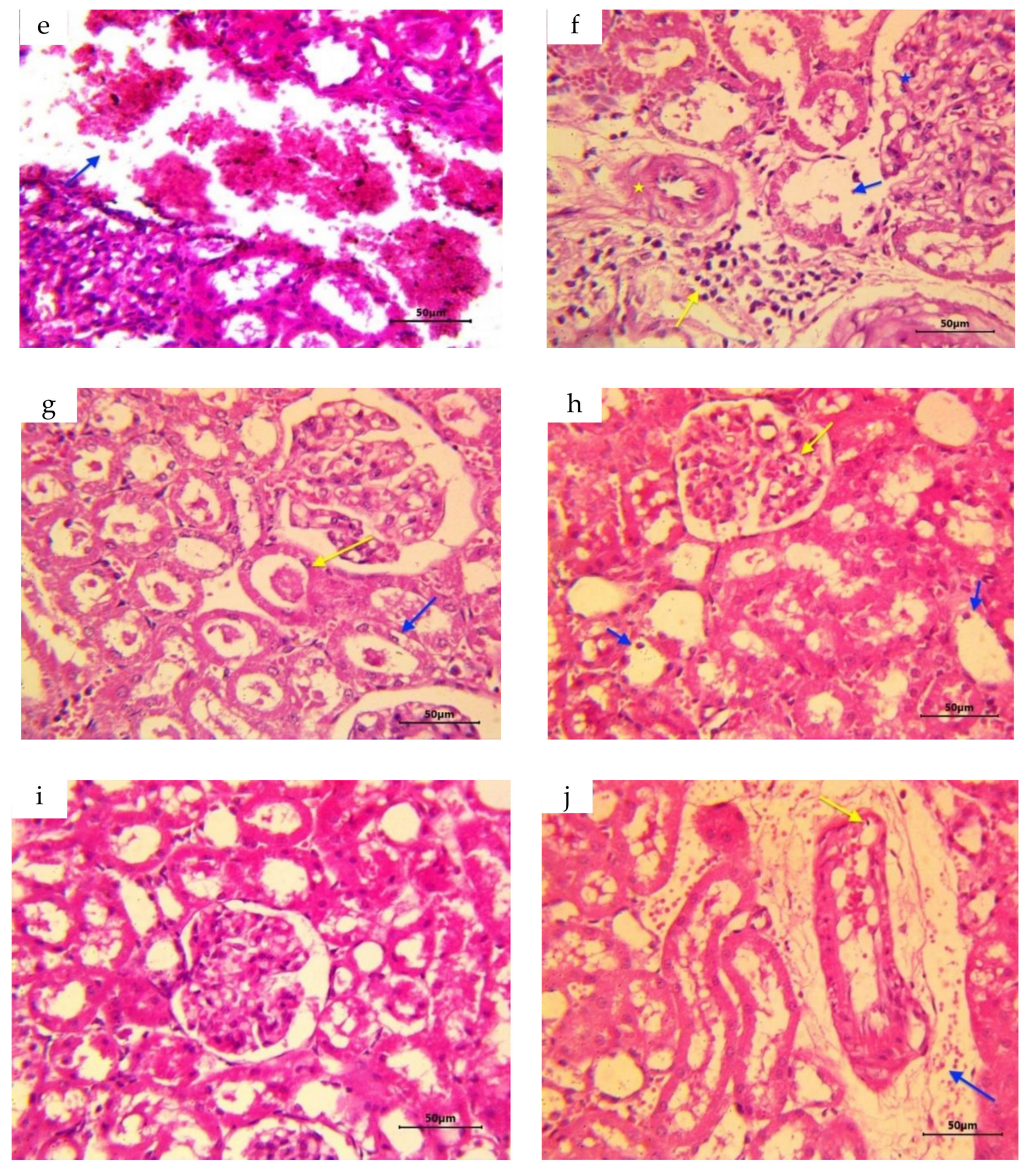
(**a**–**j**): (**a**) Group 1: Control kidney section with normal glomerulus with tuft of capillaries with Bowmen’s capsule (blue arrow) and tubules (yellow arrow) lined with cuboidal epithelium; (**b**) Group II: Normal architecture of kidney with healthy glomerulus (blue arrow) and tubules (yellow arrow); (**c**) Group III showed mild congestion (blue arrow) and mild tubular degeneration (yellow arrow); (**d**) Interstitial congestion and hemorrhage (arrow) in group IV; (**e**) Severe necrosis and interstitial hemorrhage (blue arrow) in group V; (**f**) Tubular necrosis (blue arrow) and degenerative changes in blood vessel wall (yellow star) leading to perivascular edema along with lymphoid infiltration (yellow arrow) and degenerative changes in glomerulus (blue star) in group V; (**g**) Mild vacuolar degeneration (blue arrow) in tubular epithelium and tubular casts (yellow arrow) seen in group VI; (**h**) Group VII showed mild glomerular congestion and degeneration (yellow arrow) alongside degenerative changes in tubular epithelium (blue arrows); (**i**) Group VIII showed only mild degenerative changes in glomerulus and tubular epithelium, whereas; (**j**) Group IX revealed blood vessel wall degeneration (yellow arrow) causing perivascular edema mixed with hemorrhage (blue arrow) in kidney of Wistar rats. (H&E 400X).

A comparison of lesion severity in the microscopic sections of the kidney of different groups is presented in Table 6 and Figure 3. The examination of group I sections revealed normal renal architecture, and renal tubules were lined by cuboidal epithelium. The presence of healthy glomeruli with a tuft of capillaries contained in Bowman’s capsule was noticed (Figure 4a). Microscopic sections of kidneys from rats treated with ZO extract only resembled those from the control group and appeared normal, without any pathological changes (Figure 4b). Group III rats revealed intertubular congestion, along with degeneration of the glomeruli and tubular epithelium (Figure 4c). In group IV, pathological changes such as tubular and glomerular degeneration, congestion, intertubular hemorrhage, and oedema were seen (Figure 4d). Additionally, focal areas of tubular necrosis were occasionally noticed. The maximum severity of renal changes was recorded in group V rats. Lesions in this group included widespread severe congestion, hemorrhage, and tubular degeneration, as well as tubular epithelial necrosis (Figure 4e). Randomly scattered multifocal areas of degeneration of blood vessel walls leading to perivascular edema and infiltration of inflammatory cells, mainly lymphocytes, were also observed, aside from glomerular degeneration and tubulorrhexis (Figure 4f). In group VI, microscopic examination revealed only mild changes in comparison compared to that seen in group III, which comprised mild degenerative alterations in the glomerulus and tubular epithelium, and the presence of casts in the tubular lumen alongside congestion in the interstitium (Figure 4g). Likewise, microscopic lesions in group VII were also milder in contrast to those found in group IV, comprising congestion and mild tubular as well as glomerular degeneration (Figure 4h). In addition, the presence of renal casts in the tubular lumen was also recorded. Likewise, the pathological changes observed in groups VIII and IX appeared significantly subdued compared to those seen in group V rats. In group VIII, the histopathological lesions mainly consisted of mild congestion, and mild degenerative changes in the renal tubular lining as well as the glomerulus (Figure 4i). In group IX, apart from glomerular or tubular degeneration and congestion, degenerative changes in the blood vessel wall at a few places resulting in hemorrhage and edema were also seen (Figure 4j). Moreover, occasionally, areas of tubular necrosis were observed in rats of group IX.

## 4. Discussion

Climate change coupled with intensive agricultural practices involving the non-judicious application of pesticides such as DM, as well as the increased volume of elemental pollutants, such as F^-^ in soil and groundwater, have cumulatively imposed a heavy impact on terrestrial and aquatic life forms [34,35]. Kidney cells face very high F^-^ ion concentrations, making them susceptible to F^-^ toxicity [36,37]. Exposure to DM also causes pronounced ill effects on the kidney [38]. Simultaneous exposure to multiple toxicants potentiates redox imbalance-induced renal damage compared to exposure to chemicals alone [39,40]. Urea is the end product of protein metabolism formed in the liver, whereas CR is formed from creatine after the breakdown of muscle protein, but both are excreted via kidneys, and their elevated levels indicate diminished renal functioning. Uric acid, on the other hand, is the final breakdown metabolite of purine processing, and its high serum levels point towards renal malfunction. Disturbances in electrolyte homeostasis, including disruption in the concentration of Ca and P and increased levels of BUN, CR, and UA, are considered sensitive indicators of renal malfunctioning. In the current study, all plasma renal markers were elevated in all toxicant-administered rats, which is in consonance with the earlier findings [5,38,41]. Alterations in biochemical parameters have been reported during F^-^ and arsenic exposure in previously reported studies [5,42]. Likewise, DM has been reported to induce nephrotoxicity in adult rats and their suckling pups [38]. In the present study, CR, BUN, and UA levels in the blood of co-exposed toxicant groups were significantly higher than those of individual toxicant-exposed rats, indicating greater abnormalities with co-exposure to F^-^ and DM. However, ZO extract supplementation significantly restored renal biomarkers.

Nitric oxide is a vasodilator which relaxes vascular smooth muscles, leading to an increase in blood circulation. Its levels vacillate in response to oxidative stress and inflammation in the body. Clinically, it has been reported that lower levels of NO predispose a subject to various cardiovascular diseases such as arteriosclerosis and hypotension [43,44]. A significant decrease in NO levels was observed after the administration of F^-^ or DM alone. Such reduction was more prominent after the administration of toxicants in combination. Similar findings have been documented in mice before [45]. While ZO significantly ameliorated toxicant-induced reduction in NO levels, quercetin was less effective in restoring NO levels in the co-exposure toxicant group. Exposure to F^-^ stimulates reactive oxygen production, and the resultant oxidative stress hampers antioxidant enzyme activity, as well as energy metabolism in the cells and also ion transport across plasma membranes [46]. Increased levels of NO due to the induction of induced nitric oxide synthases (iNOS), which may contribute to nitroxy radicals, induced cellular damage, while induction due to constitutive form of NOS, i.e., eNOS, has a beneficial effect on different organs and thus reduces cellular damage [44,45,46]. In the present study, the supplementation of ZO extract or quercetin enhanced the NO level and may be beneficial to the kidney by increasing the blood supply to counter toxicant-induced renal damage. Acute fatal F^-^ toxicity diminishes calcium ionic concentration in the blood, which may affect the contractability of cardiac cells, causing cardiac arrest [47]; however, prolonged exposure to F^-^ leads to electrolyte imbalance, with a significant reduction in Ca and Mg levels in plasma [48]. In the present study, the administration of DM or F^-^ decreased the plasma levels of both ions (Ca^+2^ and P), and the co-exposure of DM and F resulted in a further significant decrease. Diazinon administration in rats caused the Ca level to fall without affecting the P concentrations [49]. Derangements in blood Ca and P levels after F^-^ intoxication in rats have been previously reported [5]. Rat ameloblasts witnessed an increased Ca^+2^ influx, raising their intracellular levels upon exposure to high F^-^ concentrations [50], disturbing the balance of the Ca^+2^ to P ratio. F ions have also been reported to induce an influx of Ca^+2^ inside cells, causing drops in Ca^+2^ levels in plasma [48]. Furthermore, as the kidney is primarily responsible for F^-^ excretion, the F^-^ induced alteration in renal antioxidant enzymes in the present work might have also interfered with the absorption and renal clearance of phosphate. Since altered plasma concentration of P affects Ca^+2^ levels, it may have also contributed towards the steep fall in plasmatic Ca^+2^ concentrations. Taken together, all these could have resulted in a sharp drop in Ca^+2^ levels in blood in our work.

In the present study, the administration of F^-^ and DM alone and in combination induced a reduction in the concentrations of both enzymatic and non-enzymatic antioxidants, but increased lipid and protein peroxidation. However, these alterations were significantly reversed after ginger extract administration. GSH performs various functions in living organisms. Apart from acting as a carrier of the active thiols group due to cysteine residues present in it, it also acts as a co-factor for GST and GPx [51,52]. A decrease in the TAS spurs a decrease in parameters such as TTH, AE, and AChE. There is also strong evidence that compounds that having oxidant activity can not only modulate AChE activity but also repress its gene expression [53]. Exposure to the herbicidal agent clomazone has been shown to decrease AChE and antioxidant levels, but at the same time increase lipid peroxidation in erythrocytes [54]. Moreover, an upsurge in AOPP and MDA concentrations indicated oxidative damage to cellular proteins and lipids, respectively. Additionally, in the event of oxidative insult, the activity of AE, which grants protection to lipids against their peroxidation, may be severely curbed. Large-scale fluctuations in lipid and protein peroxidation along with the depletion of oxidant scavengers may unfold into frank tissue damage. Significantly higher depletion in levels of TAS, TTH, AE, AChE, CAT, SOD, GPx, and GR and a rise in MDA and AOPP after dual toxicant administration was seen in our study. Concurrent findings were also reported by Khan et al. [10], who studied the toxic effects of deltamethrin and F^-^ in rats. Reductions in CAT, GSH, and NO levels in rats following F^-^ toxicity were also reported [45]. Various other studies have reported a decrease in CAT, SOD, and GSH in response to pesticide and metal toxicity [14,42,55].

After the administration of toxicants, a wide variety of alterations such as degeneration, necrosis, and hemorrhage were histologically noticed in renal parenchyma in the current study, attributed most likely to toxicant-induced oxidative damage. Sharma et al. [5] also found that chronic exposure to F induced changes in oxidative stress parameters and the histomorphology of hepatic, renal, and cardiac tissues in Wistar rats. Histopathological changes were most severe in the co-administered toxicant group, which is in line with the findings of others [38,55,56]. Various experimental studies have demonstrated that simultaneous exposure to phytochemicals (flavonoids) reduces toxicant-induced renal damage [52,57,58]. In the present study, ginger administration bolstered the antioxidant profile (phytochemical ingredients) in the experimental rats. Ginger extract supplementation not only reversed deviations in plasma biomarkers of renal injury, but also corrected renal TAS, AE, AChE, GPx, GR, AOPP, and MDA levels and renal histoarchitecture in all toxicant-exposed groups, thereby authenticating the antioxidant, anti-apoptotic, and anti-inflammatory properties of ginger, as suggested by earlier studies [59]. Nephroprotection has been reported to be imparted by essential oils of ZO rhizome against cadmium toxicity in rats [60]. Similar antioxidant and chelating properties of ZO against cadmium nephrotoxicity were also described by others [61]. Essential oils from ginger have also been found to mitigate renal damage induced by the administration of cadmium and acetaminophen [60,62]. The supplementation of ginger extract effectively countered histopathological changes in renal tissues such as sloughing of tubular epithelium, dilatation of renal tubules, and interstitial fibrosis in renal parenchyma [60,62]. 6-gingerol, an active component of ginger, has been reported to efficiently alleviate kidney dysfunctions, oxidative stress, and histopathological changes induced by mercuric chloride in male rats [63]. The use of ZO in the present investigation conferred noticeable protection against the oxidative damage unleashed by dual toxicant exposure, which was better than that seen with quercetin treatment. Other studies have also validated the use of ginger as a therapeutic remedy for renal dysfunctions [64]. The result of the present study clearly demonstrates that simultaneous exposure to F^-^ and DM can cause harmful renal effects in exposed subjects. Thus, DM should be used with utmost caution as a pesticide in areas where groundwater is rich in F^-^. Furthermore, ginger supplementation can be encouraged in the population of such regions to offset kidney afflictions resulting from F^-^ and DM toxicity.

## 5. Conclusions

Observations of the study indicated that the repeated co-exposure of F^-^ and DM induced significant alterations in plasma renal biomarkers, as well as oxidative stress parameters and the histomorphology of renal tissue in Wistar rats. The changes were significantly more severe compared to those produced by exposure to an individual toxicant. The administration of ZO extract minimized F^-^- and DM-induced renal damage as indicated by improvements in the biochemical indices of renal dysfunction; reduced MDA and AOPP levels; restoration of the disturbances in the renal antioxidant system as seen by increased TTH, TAS, SOD, CAT, and GR values; and also the reversal of histomorphological changes in renal tissues in the toxicant-exposed animals.

## Figures and Tables

**Table 1:** Details of the high-performance liquid chromatography (HPLC) method.

Time (min)	Water and 0.1% Acetic Acid	Acetonitrile
0.01	95%	5%
5	95%	5%
15	10%	90%
20	10%	90%
25	95%	5%
30	95%	5%

**Table 2:** Details of the treatment regimen.

Groups	Treatment	Dose and Route of Administration
i.	Control	1 mL/day/rat, per os (PO), drinking water
ii.	*Zingiber officinale* (ZO)	300 mg/kg BW (body weight), PO
iii.	Dimethoate (DM)	31.0 mg/kg BW (1/10th LD_50_), PO
iv.	Fluoride (F^-^)	4.5 ppm in drinking water
v.	DM + F^-^	1/10th of LD_50_ (PO) + 4.5 ppm in drinking water
vi.	ZO + DM	300 mg/kg BW (PO) + 1/10th of LD_50_ (PO)
vii.	ZO + F^-^	300 mg/kg BW (PO) + 4.5 ppm
viii.	ZO + DM + F^-^	300 mg/kg BW (PO) + 1/10th of LD_50_ + 4.5 ppm
ix.	Quercetin + DM + F^-^	100 mg/kg BW (PO) + 1/10th of LD_50_ + 4.5 ppm

**Table 3:** Effect of *Zingiber officinale* (ZO) extract on subacute toxicity induced by fluoride (F^-^) and dimethoate (DM) alone and in combination on plasma renal biomarkers and minerals in Wistar rats.

Groups	BUN	CR	Uric Acid	Calcium	Phosphorus
Control	42.84 ^a^ ± 2.40	0.43 ^a^ ± 0.03	2.57 ^a^ ± 0.41	10.99 ^c^ ± 0.56	9.72 ^c^ ± 0.47
ZO Extract (300 mg/kg)	38.54 ^a^ ± 3.64	0.49 ^a^ ± 0.07	3.49 ^a^ ± 0.97	10.34 ^c^ ± 0.93	8.28 ^ab^ ± 0.67
Dimethoate (DM—1/10th LD_50_)	56.45 ^b^ ± 4.42	1.11 ^c^ ± 0.12	4.52 ^bc^ ± 0.60	5.08 ^a^ ± 0.79	5.56 ^a^ ± 0.43
Fluoride (F^-^—4.5 ppm)	55.04 ^b^ ± 2.74	1.05 ^c^ ± 0.11	3.89 ^bc^ ± 0.49	5.55 ^a^ ± 0.53	6.70 ^b^ ± 0.81
DM (1/10th LD_50_) + F^-^ (4.5 ppm)	112.99 ^d^ ± 3.69	1.50 ^d^ ± 0.08	4.82 ^c^ ± 0.47	5.21 ^a^ ± 0.84	5.32 ^a^ ± 0.59
ZO Extract + DM (1/10th LD_50_)	43.35 ^a^ ± 3.70	0.63 ^b^ ± 0.08	3.20 ^a^ ± 0.35	9.27 ^bc^ ± 0.43	9.27 ^abc^ ± 0.43
ZO Extract + F^-^ (4.5 ppm)	44.44 ^a^ ± 3.14	0.60 ^b^ ± 0.08	3.30 ^ab^ ± 0.39	10.16 ^bc^ ± 0.32	10.16 ^bc^ ± 0.32
ZO Extract + DM (1/10th LD_50_) + F^-^ (4.5 ppm)	81.37 ^c^ ± 6.58	0.53 ^ab^ ± 0.03	2.91 ^ab^ ± 0.58	12.40 ^c^ ± 0.79	10.40 ^c^ ± 0.79
Quercetin (100 mg/kg) + DM (1/10th LD_50_) + F^-^ (4.5 ppm)	72.28 ^c^ ± 9.98	0.56 ^ab^ ± 0.02	3.19 ^b^ ± 0.52	10.63 ^cd^ ± 0.72	10.63 ^cd^ ± 0.72

Values are presented as mean ± SE of 6 animals unless otherwise stated; values with different superscripts (a, b, c, d) in a column are statistically different from one another at 5% level of significance; values of BUN (blood urea nitrogen), CR (creatinine), and uric acid are expressed in mg/dL; values of calcium and phosphorus are expressed in mg/dL.

**Table 4:** Effect of *Zingiber officinale* (ZO) extract on subacute toxicity induced by fluoride (F^-^) and dimethoate (DM) alone and in combination on the renal antioxidant system in Wistar rats.

Groups	TAS	TTH	AE	AChE	CAT
Control	20.26 ^a^ ± 0.35	1.96 ^c^ ± 0.42	3.41 ^b^ ± 0.36	17,241.00 ^c^ ± 890.71	3112.69 ^b^ ± 172.21
ZO Extract (300 mg/kg)	21.68 ^a^ ± 1.25	1.98 ^c^ ± 0.36	2.96 ^b^ ± 0.25	12,907.63 ^bc^ ± 448.89	3469.04 ^b^ ± 105.89
Dimethoate (DM −1/10th LD_50_)	16.01 ^b^ ± 0.19	1.07 ^b^ ± 0.28	0.80 ^a^ ± 0.06	8269.13 ^b^ ± 302.86	3072.02 ^b^ ± 166.92
Fluoride (F^-^—4.5 ppm)	15.12 ^b^ ± 1.13	1.25 ^ab^ ± 0.54	0.79 ^a^ ± 0.04	8158.13 ^b^ ± 709.49	3187.47 ^b^ ± 586.52
DM (1/10th LD_50_) + F^-^ (4.5 ppm)	10.68 ^c^ ± 0.87	0.58 ^d^ ± 0.16	0.51 ^d^ ± 0.16	6574.50 ^a^ ± 434.04	1854.48 ^a^ ± 240.79
ZO Extract (300 mg/kg) + DM (1/10th LD_50_)	15.71 ^b^ ± 0.98	0.80 ^a^ ± 0.08	2.72 ^b^ ± 0.23	12,079.63 ^d^ ± 565.97	4372.77 ^d^ ± 898.00
ZO Extract (300 mg/kg) + F^-^ (4.5 ppm)	16.13 ^b^ ± 1.49	1.19 ^a^ ± 0.54	2.70 ^b^ ± 0.40	14,446.50 ^e^ ± 442.64	2968.29 ^b^ ± 114.96
ZO Extract (300 mg/kg) + DM (1/10th LD_50_) + F^-^ (4.5 ppm)	19.62 ^a^ ± 0.32	1.97 ^c^ ± 0.35	2.92 ^b^ ± 0.52	16,436.88 ^c^ ± 714.71	3276.49 ^b^ ± 133.96
Quercetin (100 mg/kg) + DM (1/10th LD_50_) + F^-^ (4.5 ppm)	15.53 ^b^ ± 0.86	0.90 ^a^ ± 0.24	1.47 ^a^ ± 0.28	17,569.75 ^c^ ± 962.12	3007.47 ^b^ ± 490.10

Values are presented as mean ± SE of 6 animals unless otherwise stated; values with different superscripts (a, b, c, d, e) in a column are statistically different from one another at 5% level of significance; values of TAS (total antioxidant status) are expressed in mM; values of TTH (total thiols) expressed in µM; activities of arylesterase (AE) expressed in U/mL; acetylcholinesterase (AChE) activity is expressed in nmol of thiols produced/min/mg of tissue; values of CAT (catalase) are expressed in µmol H_2_O_2_ decomposed/min/g of tissue.

**Table 5:** Effect of *Zingiber officinale* (ZO) extract on subacute toxicity induced by fluoride (F^-^) and dimethoate (DM) and in combination on the antioxidant system of renal tissue in Wistar rats.

Groups	SOD	GPx	GR	AOPP	MDA
Control	736.82 ^b^ ± 26.88	262.21 ^c^ ± 21.38	50.04 ^bc^ ± 3.26	1.42 ^a^ ± 0.14	42.03 ^a^ ± 4.55
ZO Extract (300 mg/kg)	869.32 ^b^ ± 16.46	273.16 ^c^ ± 13.08	47.30 ^b^ ± 3.41	1.51 ^a^ ± 0.05	52.15 ^a^ ± 8.98
Dimethoate (DM −1/10th LD_50_)	348.06 ^a^ ± 22.81	125.63 ^a^ ± 6.78	27.99 ^b^ ± 3.47	1.75 ^b^ ± 0.27	315.69 ^c^ ± 20.35
Fluoride (F^-^—4.5 ppm)	329.59 ^a^ ± 21.55	128.66 ^a^ ± 5.72	17.26 ^a^ ± 1.74	1.87 ^b^ ± 0.21	385.97 ^c^ ± 48.91
DM (1/10th LD_50_) + F^-^ (4.5 ppm)	252.88 ^c^ ± 11.52	193.67 ^a^ ± 1.46	14.52 ^a^ ± 1.49	2.65 ^d^ ± 0.32	492.83 ^d^ ± 15.35
ZO Extract (300 mg/kg) + DM (1/10th LD_50_)	705.58 ^b^ ± 35.99	252.26 ^bc^ ± 29.04	36.17 ^a^ ± 4.05	1.49 ^a^ ± 0.05	159.95 ^b^ ± 9.64
ZO Extract (300 mg/kg) + F^-^ (4.5 ppm)	781.95 ^b^ ± 35.79	232.84 ^b^ ± 18.99	45.12 ^bc^ ± 12.74	1.38 ^a^ ± 0.10	158.08 ^b^ ± 9.52
ZO Extract (300 mg/kg) + DM (1/10th LD_50_) + F^-^ (4.5 ppm)	738.11 ^b^ ± 24.28	205.83 ^ab^ ± 13.99	52.58 ^bc^ ± 6.87	1.44 ^a^ ± 0.04	155.74 ^b^ ± 8.06
Quercetin (100 mg/kg) + DM (1/10th LD_50_) + F^-^ (4.5 ppm)	921.80 ^d^ ± 32.34	197.89 ^ab^ ± 14.14	27.14 ^a^ ± 2.22	1.46 ^a^ ± 0.13	66.80 ^e^ ± 7.84

Values are presented as mean ± SE of 6 animals unless otherwise stated; values with different superscripts (a, b, c, d, e) in a column are statistically different from one another at 5% level of significance; values of SOD (superoxide dismutase) are expressed in Unit/g of tissue; GPx (glutathione peroxidase) is expressed in Unit/g of tissue; values of GR (glutathione reductase) are expressed nmol of NADPH/min; values of the advance oxidation protein product (AOPP) are expressed in μM of chloramine-T; values of malondialdehyde (MDA) are expressed in nmol of MDA formed/g/h.

**Table 6:** Mean histopathological lesion scores in control and different treated groups.

Treatments	Lesion Scores in Different Groups						
	Congestion	Intertubular Hemorrhage	Edema	Casts	Tubular Degeneration	Tubular Necrosis	Glomerular Degeneration
Control	0.00	0.00	0.00	0.00	0.00	0.00	0.00
ZO Extract (300 mg/kg)	0.00	0.00	0.00	0.00	0.00	0.00	0.00
Dimethoate (DM −1/10th LD_50_)	1.83 *	0.00	0.00	1.33 *	2.00 *	0.00	1.17 *
Fluoride (F^-^—4.5 ppm)	1.83 *	1.67 *	1.17 *	1.00 *	1.67 *	0.67 *	0.67 *
DM (1/10th LD_50_) + F^-^ (4.5 ppm)	2.50 **	2.33 **	2.33 **	2.17 **	2.50 **	2.17 **	2.67 **
ZO Extract (300 mg/kg) + DM (1/10th LD_50_)	1.00 *	0.00	0.00	0.83 *	1.00 *	0.00	1.17 *
ZO Extract (300 mg/kg) + F^-^ (4.5 ppm)	1.00 *	1.00 *	0.00	0.83 *	1.00 *	0.00	1.17 *
ZO Extract (300 mg/kg) + DM (1/10th LD_50_) + F^-^ (4.5 ppm)	0.67 *	0.00	0.00	1.00	0.50 *	0.00	0.83 *
Quercetin (100 mg/kg) + DM (1/10th LD_50_) + F^-^ (4.5 ppm)	0.83 *	1.33 *	1.17 *	0.83 *	1.67 *	0.83 *	0.83 *

Values with different * and ** in a column are statistically different from one another at 5% and 1% significance, respectively.

## Data Availability

All data generated for this study are contained within the article.

## Supplementary Materials

The following supporting information can be downloaded at: https://www.mdpi.com/article/10.3390/foods12091899/s1, Figure S1: Representative chromatographic fingerprinting of ZO rhizome extract (**a**) along with the standard curcumin (**b**) and quercetin (**c**).
