# Supplementary material for: Protective Effect of Quercetin and Ginger (Zingiber officinale) Extract against Dimethoate Potentiated Fluoride-Induced Nephrotoxicity in Rats
Source: Foods. 2023 May 5;12(9):1899. doi: 10.3390/foods12091899 (PMC10177764; doi:10.3390/foods12091899)
Supplement: Supplementary file 1 [file foods-12-01899-s001.zip › foods-2339802-supplementary.pdf]

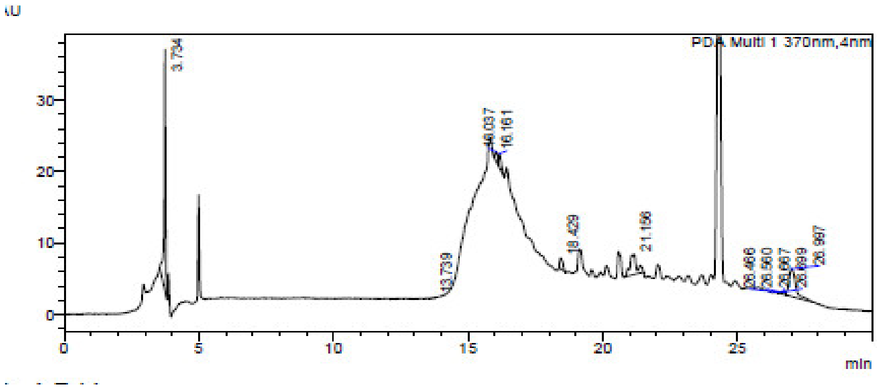

(**a**)

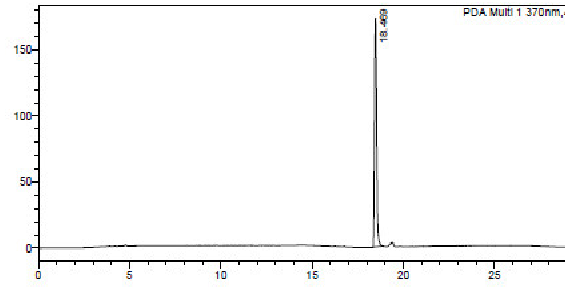

(**b**)

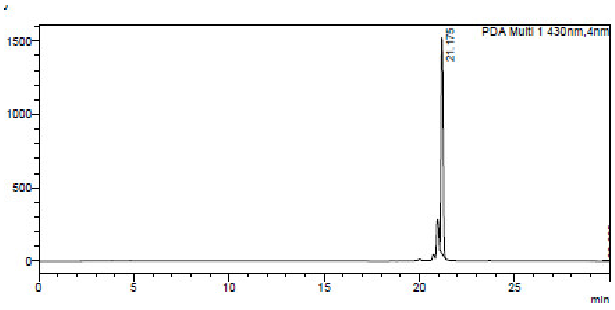

(**c**)

Figure S1. Representative chromatographic fingerprinting of ZO rhizome extract (**a**) along with the standard curcumin (**b**) and quercetin (**c**).
